# Nurses’ usage of validated tools to assess for delirium in general acute care settings: A scoping review^✰^

**DOI:** 10.1016/j.ijnsa.2026.100579

**Published:** 2026-05-26

**Authors:** Aoibhinn Boyd, Marcia Kirwan, Leona Bannon

**Affiliations:** School of Nursing Psychotherapy and Community Health, Dublin City University, Ireland

**Keywords:** Delirium, Neuropsychological tests, Nurses, Scoping review

## Abstract

**Background:**

Delirium occurs in 10–30% of hospitalised adults and is associated with an increased risk of mortality and morbidity. Best practice guidelines recommend the use of validated assessment tools for delirium. However, these tools are infrequently utilised, and delirium is routinely missed.

**Objective:**

To explore validated assessment tool usage by registered nurses in general acute care settings to assess for delirium, and the barriers and facilitators to their use.

**Methods:**

This scoping review was organised in accordance with the Joanna Briggs Institute methodology for scoping reviews. Boolean operators were used in combination with key search terms from the research questions. Sources were analysed using frequency counts and results were mapped descriptively.

**Information sources:**

CINAHL, PubMed (MEDLINE), Web of Science, Scopus, Google Scholar, and Bielefeld Academic Search Engine were searched in February 2025

**Eligibility criteria:**

Sources that provided a focus on registered nurses working in general acute care settings and their experiences using validated delirium assessment tools were considered for this scoping review.

**Results:**

Forty studies were included in this scoping review. Validated assessment tools were not used frequently by nurses to assess for delirium. Multiple barriers to using validated assessment tools were identified, with the most reported being organisational barriers. Nurses identified “opportunities to learn” as facilitators to using validated assessment tools.

**Conclusions:**

Notable heterogeneity among assessment practices were identified. Despite the availability of various validated assessment tools for delirium, usage rates remain low. Nurses have identified numerous barriers to their use, and these must be addressed to ensure accurate delirium diagnosis.

**Registration:**

This scoping review was registered on the 15th of January 2025 on Open Science Framework at https://osf.io/6qegb

**What is already known**

•Delirium is frequently undiagnosed in general acute-care settings.

•Undiagnosed delirium results in negative outcomes for a patient.

•Little is known about nurses’ utilisation of validated delirium assessment tools.

**What this paper adds**

•Highlights low validated delirium assessment tool usage rates among nurses.

•Identifies nurse reported barriers and facilitators to using such tools.

•Contributes new insights into existing variation and areas for improvement.

Alt-text: Unlabelled box dummy alt text

## 1 Background

Delirium, as defined by the Diagnostic and Statistical Manual of Mental Disorders, Fifth Edition, is an acute syndrome, which is characterised by altered levels of cognition, awareness, and attention (Diagnostic and statistical manual of mental disorders : DSM-5. Fifth edition, 2013). It is a major concern for public health, with 10–30% of hospitalised adults developing delirium (Waszynski et al., 2024). Delirium is associated with an increased risk of mortality, re-hospitalisation, falls, and skin breakdown (Waszynski et al., 2024). Additionally, postoperative delirium, a form of delirium that occurs during intensive care unit admission, is positively associated with cognitive impairments up to three years after the delirium occurs (Giordano et al., 2026). Experiencing delirium is highly distressing for many patients and this distress is mirrored in the experiences of family members and nursing staff (Devlin et al., 2018). While the exact pathophysiology of delirium remains unclear, it is hypothesised that there are numerous pathological factors that interact with each other, such as neuroinflammation or sleep deprivation, and this is what results in delirium (Inouye et al., 2014). Increased age and pre-existing dementia increase the risk of delirium exponentially. Urinary catheterisation and polypharmacy are also risk factors for delirium, as are ventilation, surgery, and increased length of hospital stay (Ahmed et al., 2014).

As delirium is a common complication in hospital settings, it is important that healthcare professionals are confidently able to prevent, diagnose, and treat delirium. As nurses provide 24-hour care to their patients, they are in a position to identify any fluctuations in cognition, awareness, or attention which indicate the need for a validated delirium assessment (Waszynski et al., 2024). Delirium is detected using assessment tools, a variety of which are available for different environments and purposes, and many of which are designed to be administered by nurses (Liu et al., 2023). However, patients do not always receive a validated delirium assessment, and frequently nurses use their personal judgement to assess for delirium or a different form of assessment rather than using a validated tool (Nydahl et al., 2024). On World Delirium Awareness Day 2023, an international study was conducted in 1664 units, investigating delirium-related clinical practices. Only 61% of delirium assessments were completed using a validated delirium assessment tool (Lindroth et al., 2024). An assessment tool is considered to be validated when it accurately identifies delirium in line with the two reference standards; the Diagnostic and Statistical Manual of Mental Disorders and the International Classification of Diseases (Lin et al., 2023). When validated tools are not used for assessment, or if assessment is not done at all, it can lead to cases of delirium not being diagnosed (Devlin et al., 2018). A systematic review of 10 studies identified that between 26–83% of delirium cases are not recognised by clinicians (Steis and Fick, 2008). Delirium remaining unrecognised can cause further harm to a patient, including increasing their risk of mortality, making efficient and effective delirium assessments essential for the wellbeing of patients (Lee et al., 2022).

There are numerous reasons for delirium not being recognised by healthcare professionals, such as the fluctuating nature of delirium, different delirium subtypes, and the similarities delirium has to dementia (Bianchi et al., 2024). Many studies have investigated nurses’ difficulties with delirium care, though few have explicitly focused on the use of assessment tools. A qualitative systematic review of 31 studies investigating nurses’ experience of delirium management identified the underutilisation and underappreciation of delirium assessment tools by nurses as a key issue surrounding delirium assessment (Lim et al., 2022). A literature review of barriers to delirium assessment also identified issues with using assessment tools as a key barrier among critical care nurses (Rowley-Conwy, 2018). Across all units and from the perspectives of various clinicians; staff shortages, lack of time to educate staff, and missing knowledge about delirium are the most reported barriers to delirium assessment (Lindroth et al., 2024). However, despite an increase in delirium research in the past number of decades, there remains significant gaps (Inouye et al., 2014; Devlin et al., 2018). While the importance of using validated assessment tools is understood, there is limited work done on the utilisation of validated delirium assessment tools in conjunction with the barriers and facilitators for the use from the perspective of nurses. Much of the literature that exists is exclusive to specific settings or contexts, such as intensive care units or postoperative delirium. This scoping review aims to provide a broad overview of the phenomenon. This review will build on what is already known about nurses' use of validated assessment tools and attempt to ascertain what is being done, what is not being done, and the reasons behind both. By doing so, gaps in assessment practice can be identified and rectified, allowing future researchers to identify methods of improving delirium care in acute care settings.

### 1.1 Aims

This scoping review aims to explore validated delirium assessment tool usage by registered nurses (nurses hereafter) in general acute care settings, and the barriers and facilitators for their use.

The research questions that will contribute to the stated aim are:

1. Is there evidence of widespread usage of validated delirium assessment tools in general acute care settings by nurses?
2. What forms of delirium assessment are most commonly utilised by nurses in general acute care settings?
3. What are the barriers and facilitators to using validated delirium assessment tools that nurses experience in general acute care settings?

## 2 Methods

### 2.1 Design

The Joanna Briggs Institute methodology for scoping reviews was used as this reviews’ framework (Peters et al., 2020). The reporting of this review used the Preferred Reporting Items for Systematic reviews Meta-Analysis extension for Scoping Reviews as a guide (Tricco et al., 2018). A protocol of this review was published and details the planned methods for this study (Boyd et al., 2025). Since the protocol, the inclusion criteria was updated to only include studies that explicitly named validated delirium assessment tools. The Network for Investigation of Delirium: Unifying Scientists website provides a standardised summary of validated delirium assessment tools. This was used as a reference list for what is considered to be a validated delirium assessment tool in this scoping review (Network for Investigation of Delirium: Unifying Scientists (NIDUS), 2018). The National Institute for Clinical Excellence delirium guidelines was used in conjunction with this list as another source of validated delirium assessment tools (National Institute for Health and Clinical Excellence, 2010). Therefore, studies that did not refer any assessment tool listed on either the website or the guidelines were excluded. Studies that did not name any specific delirium assessment tools were excluded as the validity of said tools were not defined, as were studies that only referenced non-validated forms of delirium assessment. The research question; ‘What forms of delirium assessment are most commonly utilised by nurses in general acute care settings?’ has also been updated since the protocol to include all forms of delirium assessment. While this scoping review is interested in nurses’ use of validated delirium assessment tools, it was deemed contextually important to report other valid methods of delirium assessment, such as psychiatric consultation, that do not use tools. Additionally, for studies that met the inclusion criteria, any non-validated methods of delirium assessment were captured as complementary information. Various, non-validated, methods of delirium assessment do not involve the use of tools, such as observation or personal judgement. By reframing the research question, these methods of delirium assessment can be captured within studies that met the inclusion criteria. An extensive literature search was conducted in Joanna Briggs Institute Evidence Synthesis, and The Cochrane Database of Systematic Reviews, Scopus, CINAHL, and PubMed (MEDLINE) and no similar scoping or systematic reviews were identified.

### 2.2 Search methods

Using the key words from the established research questions, an initial search was carried out on CINAHL and PubMed (MEDLINE) and relevant articles were identified. With the support of a Dublin City University subject librarian, the search strategy was developed using the identified article titles. The search strategy developed for PubMed can be found in Table 1**.** CINAHL, PubMed (MEDLINE), Web of Science, and Scopus were the databases used for this scoping review. Google Scholar and Bielefeld Academic Search Engine were used to search grey literature. Databases were searched on the 19th of February 2025. The citation lists of included sources were searched for additional sources by the research team in July 2025.

**Table 1:** Search strategy table for PubMed.

Search No.	Free Text Search Terms
S1	Delirium OR “acute confusional state” OR encephalopathy OR “cognitive disorder” OR “acute confusion” OR “disorientation”
S2	Instrument OR exam* OR scale
S3	Assess* OR screen* OR diagno* OR detect
S4	S2 AND S3
S5	4AT OR “confusion assessment” OR CAM OR AMT
S6	S4 OR S5
S7	Nurs*
S8	S1 AND S6 AND S7

### 2.3 Inclusion and exclusion criteria

The full inclusion and exclusion criteria can be found in Table 2.

**Table 2:** Inclusion and exclusion criteria.

	Inclusion	Exclusion
**Population**	Sources that report on registered nurses working in general acute hospitals	Sources that do not explicitly report on registered nurses working in general acute hospitals
**Concept**	Sources that primarily examine delirium validated assessment tools and make explicit reference to a validated delirium assessment tool Sources using a pre-test/post-test design reporting information regarding barriers and facilitators to validated assessment tool use	Sources that do not make an explicit reference to a delirium validated assessment tool Sources using a pre-test/post-test design that does not report information regarding barriers and facilitators to validated assessment tool use
**Context**	Sources that explicitly report on nurses who are employed in an adult ward in a general acute hospital	Sources that do not explicitly report on nurses who are employed in an adult ward in a general acute hospital
**Types of sources**	Types of sources will be left open to include both peer-reviewed studies and grey literature	Duplications of sources already included
**Language**	Sources that are available in English	Sources that are not available in English
**Time Period**	Sources produced between 2000 and 2025	Sources produced before 2000

Population:

The population of this scoping review is registered nurses and therefore, for a study to be considered for inclusion, registered nurses must have been participants. If a study did not investigate nurses exclusively, the results from the nurse participants must have been explicit to be considered for inclusion. Studies that solely investigated student nurses and other healthcare workers were excluded.

Concept:

The concept of interest for this review is the use of validated assessment tools for delirium in hospitals. Studies that examine nurses’ experiences using validated tools were considered, including the types of tools used, the rates of usage, and the barriers and facilitators to using the tools. While other methods of delirium assessment were captured in the results, an explicit reference to a validated delirium assessment tool was required for a study to be considered for inclusion. This was to ensure that the concept of the review remained focused on validated delirium assessment tools, and other methods of assessment were captured as complementary information. Studies that investigated rates of assessment tool usage at multiple time points, such as pre-test and post-test designs, were excluded. Investigating the impact of interventions, whether that be educational programmes or protocol implementations, is outside the scope of this review. The rates of assessment tool usage in these studies are influenced by the study intervention and therefore not reflective of standard contexts. For this reason, these study types were excluded, unless they provided information on nurse identified barriers and facilitators to assessment tool usage. Information regarding barriers and facilitators from these study designs were included as the impact the intervention had on barriers or facilitators is within the scope of the review. Translation or validation studies were not included as they do not address the research question.

Context:

This review considered sources primarily centred on adult, general acute care hospital units. Nurses working in paediatric units, long-term care facilities, nursing homes, or palliative care units are beyond the scope of this review. If a study did not investigate general acute care settings exclusively, the results from the general acute care settings must have been explicit to be considered for inclusion. There was no geographical limit to this review.

### 2.4 Selection of sources

All study methodologies were considered for this review, including quantitative and qualitative study designs. Grey literature, such as reports and academic theses were also considered. Only sources published in English were considered for inclusion as translation of other sources was not feasible for the research team. Only sources published between the years 2000 and 2025 were considered. Prior to the year 2000, terminology in delirium research was broad and inconsistent, creating barriers to cohesive research. However, the publication of the Diagnostic and Statistical Manual of Mental Disorders, Fourth Edition, Text Revision in 2000 improved the standardization of delirium research terminology (Morandi et al., 2008). Only sources published in 2025 and before were considered as this is when the database search took place.

### 2.5 Search outcome

Sources identified using the search strategy were uploaded into Covidence™. Two researchers independently screened the title and abstracts of sources using the inclusion criteria. In line with Joanna Briggs Institute guidelines, a pilot test of source selectors was conducted prior to full-text screening (Peters et al., 2020). This included the full research team screening a random sample of sources against the eligibility criteria in order to ensure minimal discrepancies occurred. After the pilot test was complete, potentially relevant sources were independently assessed in detail by two researchers also using the inclusion criteria. Reasons for exclusion were recorded for excluded sources. Any conflicts in agreement between the researchers were solved through discussion and the input of a third researcher.

### 2.6 Data extraction

A data extraction tool was developed by the research team. To ensure the tool was effective, the tool was piloted by the research team on 10% of included studies. Key information regarding the source (name of the authors; year of publication; country study was conducted; study type; and study methodology), the population sample, the delirium assessment tools investigated, and barriers and facilitators to the tool were extracted. The data extraction tool can be found in supplementary file 1.

### 2.7 Analysis and presentation of results

In line with the aim of scoping reviews, the results are mapped descriptively (Arksey and O’Malley, 2005). The results of the search are presented using a Preferred Reporting Items for Systematic reviews Meta-Analysis extension for Scoping Reviews flow diagram. A descriptive summary of all extracted data is presented in the form of a table followed by a description of results through a narrative summary.

## 3 Results

**Fig. 1:**
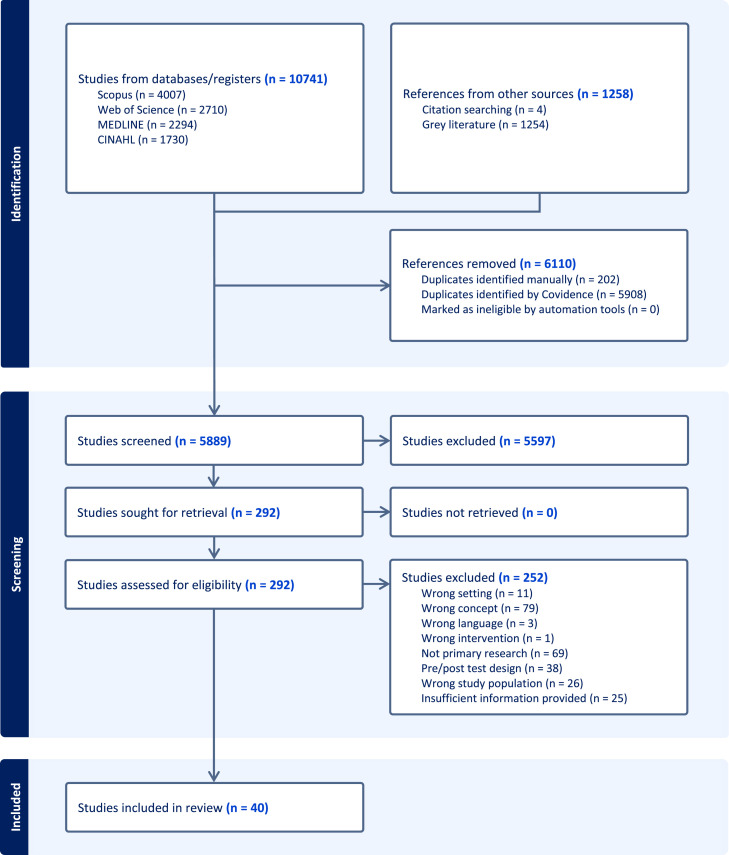
PRISMA-ScR flow Fig. 1. PRISMA-ScR flow diagram illustrates the search and selection of sources used in ‘nurses’ usage of validated tools to assess for delirium in general acute care settings: a scoping review’. Within the figure, the quantity of sources identified from each database, duplicates removed, titles/abstracts screened, full-text articles assessed for eligibility, and final studies included in the review are illustrated. Reasons for exclusion at the full-text stage are also included.

After duplications were removed, 5886 studies remained. Title and abstract screening removed an additional 5597 studies. The remaining studies underwent a full-text review, resulting in thirty-eight studies for inclusion. The most common reasons for exclusion at this stage were studies having the wrong concept (*n* = 79), studies not being primary research (*n* = 69), and studies having a pre/post-test design (*n* = 38). An additional two studies were identified in the forward and backward citation process and included at the full text stage, totalling forty studies to be included in this scoping review. Fig.1 details the Preferred Reporting Items for Systematic reviews Meta-Analysis extension for Scoping Reviews flow diagram.

### 3.1 Characteristics of included studies

Included studies were published between the years 2008 and 2025. Studies were conducted in a variety of countries, the most common being the USA conducting a total of eight studies. To answer their own specific research questions, included studies used a variety of methodologies and a diverse range of study designs. Twenty-two studies used a quantitative methodology, twelve studies used a qualitative methodology, and six studies used a mixed methods methodology. Thirty-five studies were journal articles, however as this scoping review also included a grey literature search, three thesis manuscripts and two abstracts were included. These outliers are highlighted beside study methodology in Table 3. As per the inclusion criteria, all studies included nurses as a population group and sample sizes ranged from 7 to 616. All studies contained results based in general acute care settings as required by the inclusion criteria. Twenty-two studies were exclusively set in critical care units. Some studies investigated multiple units including units that were in the exclusion criteria, such as emergency departments. Only data from units that reached the inclusion criteria was extracted from these studies. Eighteen studies examined nurses’ usage of validated assessment tools, and thirty-five studies examined the barriers and facilitators for the tools’ use. Table 3 outlines the characteristics and results of the included studies.

**Table 3:** Results of included studies.

First author (year), country study was conducted	Study methodology	Nurse sample size	Setting results were extracted from	Nurses validated tool usage	Validated delirium assessment tools:	Other reported methods of delirium assessment:	Barriers	Facilitators
Aldwikat (2023), Australia	Qualitative study: Focus group design	24	Post anaesthesia care unit, orthopaedic surgical units	Not reported	3D-Confusion Assessment Method 4AT Delirium Observation Screening Scale	Glasgow Coma Scale	Tool is considered unnecessary Fear of harming patient Patient factors make tool harder to complete Patient and families disrupt the use of the tool Nurses are at their capacity No organisational supports for the use of tools	Ease and efficiency of tool Positive staff culture
Alhaidari (2017), New Zealand	Mixed methods study: sequential-explanatory design	12	Medical units	Not reported	Short Confusion Assessment Method	Not reported	Mistrust in tool No organisational supports for the use of tools	Not reported
Anbu (2014), Ireland	Quantitative study: descriptive design (Masters Thesis)	110	Critical care units	60.6% of nurses use Confusion Assessment Method for the Intensive Care Unit once or more per 12-hour shift 19.2% of nurses use Intensive Care Delirium Screening Checklist once or more per 12-hour shift	Confusion Assessment Method for the Intensive Care Unit Intensive Care Delirium Screening Checklist	Ability to follow commands Clinical Institute Withdrawal Assessment of Alcohol Scale, Revised Evaluation of agitated related events Glasgow Coma Scale Psychiatric consultation Other	Difficulty and complexity of tool	Not reported
Andrews (2015), USA	Mixed methods study: Two-group pretest-post-test design	20	Critical care unit	Not reported	Confusion Assessment Method for the Intensive Care Unit	Not reported	Nursing beliefs Patient factors make tool harder to complete Negative culture towards tools	Ease and efficiency of tool Opportunities to learn
Birge (2024), Turkey	Quantitative study: Cross-sectional design	181	Oncology units	5.5% of nurses use a screening tool in delirium assessment Of nurses that use screening tools, 10% use Confusion Assessment Method*	Confusion Assessment Method	Glasgow Coma Scale Richmond Agitation-Sedation Scale Recognition of delirium symptoms	Not reported	Not reported
Biyabanaki (2020), Iran	Quantitative study: Cross-sectional design	167	Critical care units	24.4% of nurses never use Confusion Assessment Method for the Intensive Care Unit for delirium assessment 33.3% of nurses never use Intensive Care Delirium Screening Checklist for delirium assessment	Confusion Assessment Method for the Intensive Care Unit Intensive Care Delirium Screening Checklist	Ability to follow commands Evaluation of agitated related events Psychiatric consultation	Difficulty and complexity of tool Nursing beliefs	Not reported
Carin-Levy (2013), UK	Quantitative study: Cross-sectional design	29	Specialist stroke units and general units	3.4% of nurses exclusively use a validated assessment tool 27.5% use both validated assessment tools and clinical judgement 24.1% of all nurses use Confusion Assessment Method 13.7% of all nurses use ‘other’ tool	Confusion Assessment Method	Clinical judgement Other	No organisational supports for the use of tools	Not reported
Correya (2025), Australia	Qualitative study: exploratory descriptive design	23	Critical care unit	Not reported	Confusion Assessment Method for the Intensive Care Unit	Not reported	Lack of knowledge and skills Patient factors make tool harder to complete Patient and families disrupt the use of the tool Negative culture towards tools No organisational supports for the use of tools	Improved understanding of delirium Opportunities to learn Positive staff culture
Devlin (2008), USA	Quantitative study: Cross-sectional design	331	Critical care units	36% of nurses use Confusion Assessment Method for the Intensive Care Unit once or more per 12-hour shift 11% of nurses use Intensive Care Delirium Screening Checklist once or more per 12-hour shift	Confusion Assessment Method for the Intensive Care Unit Intensive Care Delirium Screening Checklist	Ability to follow commands Clinical Institute Withdrawal Assessment of Alcohol Scale, Revised Evaluation of agitated related events Psychiatric consultation	Difficulty and complexity of tool	Not reported
dosSantos (2022), Brazil	Qualitative study: Descriptive design	32	Critical care units	Not reported	Confusion Assessment Method for the Intensive Care Unit	Not reported	Lack of knowledge and skills Patient factors make tool harder to complete Patient and families disrupt the use of the tool No organisational supports for the use of tools	Not reported
Emme (2020), Denmark	Qualitative study: Descriptive design	23	Geriatrics unit, endocrinology unit, cardiology unit, surgical unit	Not reported	Brief Confusion Assessment Method Confusion Assessment Method	Not reported	Difficulty and complexity of tool Tool is considered unnecessary Lack of knowledge and skills Nursing beliefs Patient and families disrupt the use of the tool Nurses are at their capacity No organisational supports for the use of tools	Ease and efficiency of tool
Fraser (2018), Canada	Mixed methods study: Pre-post study design	30 (survey) 4 (focus groups)	Surgical unit	Not reported	Intensive Care Delirium Screening Checklist	Not reported	Lack of knowledge and skills	Improved understanding of delirium
Gao (2022), China	Quantitative study: Cross-sectional design	237	Critical care units	51.5% of nurses use assessment tools to assess for delirium Of nurses that use assessment tools, 58.1% use Confusion Assessment Method for the Intensive Care Unit, 9.5% use Intensive Care Delirium Screening Checklist	Confusion Assessment Method for the Intensive Care Unit Intensive Care Delirium Screening Checklist	Clinical judgement Doctor consultation Richmond Agitation-Sedation Scale	Tool is considered unnecessary Lack of knowledge and skills No organisational supports for the use of tools	Not reported
HallbergKristensen (2024), Sweden	Qualitative study: descriptive design with an inductive approach	12	Cardiac surgical unit	Not reported	Nursing Delirium Screening Scale	Not reported	Difficulty and complexity of tool Length of tool Negative culture towards tools Nurses are at their capacity	Not reported
Jung (2013), Korea	Qualitative study: Qualitative research design	18	Critical care unit	Not reported	Confusion Assessment Method for the Intensive Care Unit	Not reported	Fear of harming patient Patient factors make tool harder to complete Negative culture towards tools Hospital environment	Opportunities to learn
Kandindi (2018), South African	Quantitative study: Cross-sectional design (Master’s Thesis)	100	Critical care units	24% of nurses use Confusion Assessment Method for the Intensive Care Unit once or more per 12-hour shift 15% of nurses use Intensive Care Delirium Screening Checklist once or more per 12-hour shift	Confusion Assessment Method for the Intensive Care Unit Intensive Care Delirium Screening Checklist	Ability to follow commands Clinical Institute Withdrawal Assessment of Alcohol Scale, Revised Evaluation of agitated related events Psychiatric consultation	Nursing beliefs	Not reported
Lange (2023), Poland	Quantitative study: Cross-sectional design	382	Critical care units	24% of nurses use Confusion Assessment Method for delirium assessment	Confusion Assessment Method	Ability to follow commands Clinical Institute Withdrawal Assessment, Alcohol, Revised Evaluation of agitated related events Psychiatric consultation Screening checklist	Nursing beliefs	Not reported
Law (2012), USA	Quantitative study: Cross-sectional design	84	Oncology units	Not reported	Intensive Care Delirium Screening Checklist	Not reported	Negative culture towards tools Nurses are at their capacity	Not reported
Lunghi (2012), USA	Quantitative study: Cross-sectional design (Poster Abstract)	35	Critical care unit	46.4% of nurses screen >75% of patients using Confusion Assessment Method for the Intensive Care Unit	Confusion Assessment Method for the Intensive Care Unit	Not reported	Patient factors make tool harder to complete	Not reported
Meghani (2024), Ireland	Quantitative study: Descriptive exploratory design	103	Critical care unit	14% of nurses use Confusion Assessment Method for the Intensive Care Unit or Intensive Care Delirium Screening Checklist as part of current practice	Confusion Assessment Method for the Intensive Care Unit Intensive Care Delirium Screening Checklist	Not reported	Difficulty and complexity of tool Length of tool Lack of knowledge and skills	Not reported
Nydahl (2018), German speaking countries	Quantitative study: Cross-sectional design	482	Critical care units, operation theatres, anaesthesia units, intermediate care units	Over 50% of nurses use validated assessment tools for delirium assessment	Confusion Assessment Method for the Intensive Care Unit Intensive Care Delirium Screening Checklist Nursing Delirium Screening Scale	Observation of suspect behaviour Other	Not reported	Not reported
Oberai (2019), Australia	Qualitative study: in-depth focus groups	14	Orthopaedic unit	Not reported	4AT	Not reported	Tool is considered unnecessary Nursing beliefs Negative culture towards tools	Improved understanding of delirium
Oxenbøll-Collet (2018), Denmark	Qualitative study: explorative multicentre design	20	Critical care units	Not reported	Confusion Assessment Method for the Intensive Care Unit	Not reported	Length of tool Mistrust in tool Nursing beliefs Fear of harming patient Patient factors make tool harder to complete Patient and families disrupt the use of the tool Negative culture towards tools	Not reported
Özsaban (2016), Turkey	Quantitative study: Descriptive, correlational study design	301	Critical care units	Of the 67.8% of nurses who perform routine delirium assessments, 6.3% use Nursing Delirium Screening Scale, 4.4% use Confusion Assessment Method for the Intensive Care Unit, 4% use Intensive Care Delirium Screening Checklist	Confusion Assessment Method for the Intensive Care Unit Intensive Care Delirium Screening Checklist Nursing Delirium Screening Scale	Psychiatric consultation	Difficulty and complexity of tool	Not reported
Pinto (2016), Italy	Quantitative study: Cross-sectional design	108	Critical care units	48% of nurses do not use the Confusion Assessment Method for the Intensive Care Unit (and Richmond Agitation-Sedation Scale) in daily practice**	Confusion Assessment Method for the Intensive Care Unit	Richmond Agitation-Sedation Scale	Not reported	Not reported
Ragheb (2023), USA	Qualitative study: Pre-implementation diagnostic evaluation design	18	Medical-surgical units	Not reported	Confusion Assessment Method	Not reported	Tool is considered unnecessary Negative culture towards tools Nurses are at their capacity No organisational supports for the use of tools	Establishing baseline mental status Opportunities to learn
Ramoo (2018), Malaysia	Quantitative study: Single-group pretest-post-test design	61	Critical care unit	Not reported	Confusion Assessment Method for the Intensive Care Unit	Not reported	Difficulty and complexity of tool Length of tool Patient factors make tool harder to complete Negative culture towards tools	Not reported
Reade (2011), Australia	Quantitative study: Pretest-post-test design (Poster Abstract)	65	Critical care unit	Not reported	Confusion Assessment Method for the Intensive Care Unit	Not reported	Difficulty and complexity of tool Mistrust in tool	Not reported
Reppas-Rindlisbacher (2021), Canada	Quantitative study: Cross-sectional design	50	Internal medicine units	Not reported	Confusion Assessment Method	Not reported	Fear of harming patient Patient factors make tool harder to complete Hospital environment Nurses are at their capacity No organisational supports for the use of tools	Establishing baseline mental status
Rowley-Conwy (2017), UK	Quantitative study: Survey-questionnaire design	31	Critical care unit	39% of nurses use Confusion Assessment Method for the Intensive Care Unit for delirium assessment	Confusion Assessment Method for the Intensive Care Unit	Clinical judgement	Difficulty and complexity of tool	Not reported
Scott (2013), UK	Quantitative study: Single centre service evaluation design	72 (primary survey) 47 (follow-up survey)	Critical care unit	Not reported	Confusion Assessment Method for the Intensive Care Unit	Not reported	Patient factors make tool harder to complete Negative culture towards tools	Not reported
Soja (2008), USA	Mixed methods study: Prospective, observational study design	42	Trauma unit	Not reported	Confusion Assessment Method for the Intensive Care Unit	Not reported	Lack of knowledge and skills Nurses are at their capacity	Not reported
Steinseth (2018), Norway	Mixed methods study: Explanatory sequential design.	7	Critical care unit	Not reported	Confusion Assessment Method for the Intensive Care Unit	Not reported	Tool is considered unnecessary Mistrust in tool Fear of harming patient No organisational supports for the use of tools	Opportunities to learn Positive staff culture
Swan (2011), USA	Qualitative study: descriptive design	15	Orthopaedic units	Not reported	Confusion Assessment Method	Not reported	Tool is considered unnecessary Nursing beliefs Fear of harming patient Patient factors make tool harder to complete Nurses are at their capacity No organisational supports for the use of tools	Establishing baseline mental status Opportunities to learn
Trask (2021), USA	Mixed methods study: Cross-sectional multi-methods design (PhD Thesis)	85	Critical care unit	95.3% of nurses use Confusion Assessment Method for the Intensive Care Unit for delirium assessment*** 56.5% of nurses use the Confusion Assessment Method for the Intensive Care Unit once per 12-hour shift 38.8% of nurses use Confusion Assessment Method for the Intensive Care Unit once or more per 12-hour shift	Confusion Assessment Method for the Intensive Care Unit	Ability to follow commands Evaluation of orientation level Psychiatric consultation Richmond Agitation-Sedation Scale	Patient factors make tool harder to complete	Not reported
Wong (2018), Canada	Qualitative study: focus group design	43	Orthopaedic units	Not reported	Confusion Assessment Method	Not reported	Lack of knowledge and skills Nursing beliefs Patient factors make tools harder to complete Negative culture towards tools Nurses are at their capacity No organisational supports for the use of tools	Ease and efficiency of tool
Yang (2024), China	Quantitative study: Cross-sectional design	616	Orthopaedic units	Of the 26.79% of nurses who screen for delirium, 59.39% use Confusion Assessment Method, 22.42% use Nursing Delirium Screening Scale, 18.18% use 4AT	4AT Confusion Assessment Method Nursing Delirium Screening Scale	Not reported	Not reported	Not reported
Yuan (2022), China	Quantitative study: Cross-sectional design	213	Post anaesthesia care units	30.2% use Confusion Assessment Method for delirium assessment 19.7% use Clinical Assessment of Confusion for delirium assessment 15.4% use Bedside Confusion Scale for delirium assessment 11.2% use Cognitive Test for Delirium for delirium assessment 5.6% use Clinical Global Impressions Scale Delirium for delirium assessment	Bedside Confusion Scale Confusion Assessment Method Clinical Assessment of Confusion Cognitive Test for Delirium Clinical	Recognition of delirium symptoms Global Impressions Scale Delirium	Not reported	Not reported
Zamoscik (2017), UK	Qualitative study: focus group design	12	Critical care unit	Not reported	Confusion Assessment Method for the Intensive Care Unit	Not reported	Mistrust in tools Lack of knowledge and skills Nursing beliefs Patient factors make tools harder to complete Patient and families disrupt the use of the tools Negative culture towards tools Nurses are at their capacity	Opportunities to learn
Zhou (2023), China	Quantitative study: Cross-sectional design	477	Critical care units, orthopaedic units, surgical units, anaesthesiology units, geriatric units	31.45% of nurses use validated tools for delirium assessment Of nurses that use validated assessment tools, 37.33% use Confusion Assessment Method, 28% use Confusion Assessment Method for the Intensive Care Unit, 12.67% use Intensive Care Delirium Screening Checklist, 8.67% use Nursing Delirium Screening Scale, 13.33% use others	Confusion Assessment Method Confusion Assessment Method for the Intensive Care Unit Intensive Care Delirium Screening Checklist Nursing Delirium Screening Scale	Clinical judgement Other	Lack of knowledge and skills No organisational supports for the use of tools	Not reported

* 10% was extrapolated as out of the 10 nurses that use screening tools, 1 uses confusion assessment method.

**Data collected includes richmond agitation-sedation scale use by nurses.

***Figure was extrapolated by combining 56.5% and 38.8%.

### 3.2 Widespread use of validated assessment tools

Eighteen studies collected numerical data on nurses’ validated assessment tool usage (see Table 3). Validated assessment tool usage ranged from 95.3% (Trask, 2021) to as low as 0.55% (Birge and Kutlutürkan, 2024). It is not possible to accurately compare the different rates provided in the studies due to the heterogeneity among studies. Some studies allowed participants to select multiple assessment tools to indicate their varied usage while other studies used binary questions to investigate if nurses used validated assessment tools. Some studies reported delirium assessment tool usage as a percentage of all participants, while other studies reported usage as a percentage of participants who routinely screen for delirium. With regards to what is considered ‘use’ of validated assessment tools, there is also substantial variety. Some studies do not discuss frequency of tool use, other studies categorise tool use by ‘never’, ‘rarely’, ‘once per shift’ etc. This renders the studies incomparable as there is not a baseline frequency that constitutes ‘use’ of the tools. What determines widespread use of validated assessment tools can therefore not be determined. However, it is clear from the included studies that there are low usage rates of validated tools among nurses. In the majority of studies, less than half of participants use validated tools to assess for delirium.

### 3.3 Commonly used forms of delirium assessment

All forty studies identified one or more forms of validated delirium assessment. Some studies measured the rate of different tool usage while others identified the tool while discussing barriers and facilitators. The Confusion Assessment Method for the Intensive Care Unit was used in some capacity in twenty-three of the studies and the Intensive Care Delirium Screening Checklist was used in eleven of the studies (see Table 3). Both the Confusion Assessment Method for the Intensive Care Unit and the Intensive Care Delirium Screening Checklist are the recommended tools for intensive care unit delirium assessment, and the majority of studies were set exclusively in critical care units. Moving out of the critical care setting, the Confusion Assessment Method and the Nursing Delirium Screening Scale were the most commonly used tools for delirium assessment, the Confusion Assessment Method being used in eleven studies and the Nursing Delirium Screening Scale being used in five studies (see Table 3). Included studies report a variety of delirium assessments that do not use validated assessment tools. Psychiatric consultation was used in six studies, the ability to follow commands was used in five studies, evaluation of agitated related events was used in four studies and clinical judgement was used in four studies (see Table 3). In addition to this, Clinical Institute Withdrawal Assessment of Alcohol Scale, Revised was used in four studies and Richmond Agitation-Sedation Scale was used in four studies (see Table 3). While these are validated tools, they are not validated for the assessment of delirium.

### 3.4 Barriers to using validated delirium assessment tools

A total of fifty-six barriers were identified within thirty-five studies in this scoping review. Individual barriers were grouped by similar concepts which are reported in Table 3. For this narrative summary, concepts are divided into four themes: barriers at the assessment tool level, barriers at the individual nurse level, barriers at the patient level, and barriers at the organisational level. A full list of barriers and corresponding themes can be found in supplementary file 2.

Barriers at the assessment tool level refer to complaints and problems nurses have about the specific validated delirium assessment tools. Nurses dislike the assessment tools and have negative attitudes and opinions towards them. In ten studies, nurses deem assessment tools to be too difficult and too complex. Five studies highlighted a lack of trust in the tools and the results produced. Six studies identified that tools are deemed unnecessary, either because the tools themselves are not adequate or because the existing documentation nurses complete is sufficient in the assessment of delirium (see Table 3). The perceived long length of time assessment tools take is also considered a barrier to use in four of the studies (see Table 3).

Barriers at the individual nurse level were the least cited within the studies. These barriers refer to deficits within the nurses’ own knowledge or abilities that in turn create barriers to assessment. In ten studies, nurses report that they lack the knowledge and the skills necessary to use validated delirium assessment tools (see Table 3). The lack of knowledge refers to both knowledge about delirium and knowledge about the tools themselves. Lack of skills not only refers to skills to use the tools but also the ability to differentiate between delirium and other conditions. Equally, in ten studies, nurses have beliefs that create a barrier to using the tool, such as a lack of confidence in their own ability to use a tool, not considering delirium assessments to be a priority or not believing it is the nurses’ role to diagnose (see Table 3).

Barriers at the patient level refer to barriers created by the patients, albeit inadvertently. In fourteen studies, these barriers relate to patient factors that make the use of tools difficult (see Table 3). These include patients who do not speak the same language as the nurses, patients who cannot understand the assessment tool questions, and intubated or sedated patients. In addition to these patient factors, in six studies, patients and their families were noted as being disruptive to the use of the tool, such as when patients resist assessments (see Table 3). Conversely, concern for the patient was also cited as a barrier. In six studies nurses fear that using assessment tools can harm the patient (see Table 3). Using assessment tools was considered to burden the patient, particularly when they are regularly repeated.

Barriers at the organisational levels were the most commonly cited by nurses. These barriers refer to organisational structures within the general acute care setting that impede the use of validated assessment tools. Negative staff culture towards assessment tools was discussed in thirteen studies, specifically that the findings from the assessment tools are not valued by other staff (see Table 3). The lack of organisational support for the use of tools was also discussed in thirteen studies (see Table 3). Nurses cite lack of training as a substantial barrier to the use of assessment tools as well as the difficulty they have establishing a patient's baseline mental status. In ten studies nurses indicate that they are at their capacity and time constraints are a barrier to the use of assessment tools (see Table 3). Finally, two studies indicate that the general hospital environment makes the use of assessment tools a challenge (Jung et al., 2013; Reppas-Rindlisbacher et al., 2021). The environment is often loud, nurses may need to wear gloves or masks, and there is a lack of suitable interpretation services for patients who need them.

### 3.5 Facilitators to using validated delirium assessment tools

A total of twenty facilitators were identified within thirteen studies. The facilitators have been grouped into five themes; ease and efficiency of tools, opportunities to learn, improved understanding of delirium, establishing baseline mental status, and positive staff culture. These themes are reported in Table 3. A full list of facilitators and corresponding themes can be found in Supplementary file 3.

The ease and efficiency of tools were highlighted by nurses as facilitators in four studies (see Table 3). Nurses noted that when the assessment tool was integrated into their current tasks, it was easier to remember and efficiently complete.

Opportunities to learn were the most cited facilitators by nurses in seven studies (see Table 3). Nurses expressed that opportunities to receive education, training, and practice facilitate their use of the tool. The existence of a delirium assessment protocol within their workplace was also cited as a way of facilitating the use of delirium tools.

Three studies claim having an improved understanding of delirium acts as a facilitator to the use of delirium assessment tools (Fraser et al., 2018; Oberai et al., 2019; Correya et al., 2025). Unlike opportunities to learn, these facilitators are not necessarily provided by the hospital. Having a general awareness and having existing knowledge about delirium facilitates the use of the assessment tool. Additionally, understanding the benefits of using an assessment tool also encourages nurses to use the tool more frequently.

Methods of establishing a patient’s baseline mental status was discussed as facilitators to assessment tool use in three studies (Swan et al., 2011; Reppas-Rindlisbacher et al., 2021; Ragheb et al., 2023). Options for this were pre-screening all patients before the need for delirium assessments or promoting the involvement of family members who can provide the nurses with a baseline mental status.

Having a positive staff culture was considered to be a facilitator to assessment tool use in three studies (Steinseth et al., 2018; Aldwikat et al., 2023; Correya et al., 2025). This referred to both having supportive and open-minded staff and having encouragement and guidance from leadership.

## 4 Discussion

The aim of this scoping review was to explore validated delirium assessment tool usage by nurses in general acute care settings, and the barriers and facilitators for their use. To achieve this aim, the authors investigated if there is widespread usage of validated delirium assessment tools, what forms of delirium assessment are most commonly utilised, and what barriers and facilitators to using validated delirium assessment tools nurses experience. To the authors’ knowledge, this is the first scoping review to examine this specific topic. The results of the review revealed generally low usage of validated delirium assessment tools among nurses in general acute care settings, the most frequently reported validated tool was the Confusion Assessment Method for the Intensive Care Unit, the most commonly identified barriers to using validated tools were at the organisational level, and opportunities to learn were the most commonly identified facilitators. A total of forty studies were included in this scoping review. While studies published after the year 2000 were considered, only studies published in 2008 and onwards were deemed suitable. The majority of studies included were published during or after 2018, highlighting an increase in delirium research in recent years. While there was a diverse range of units in which studies were conducted, the majority of studies were conducted in critical care units. As delirium is a more common occurrence in critical care units than in other units, it is logical that a high proportion of studies focus on this setting (Inouye et al., 2014).

In the majority of included studies in this review, where rates of usage are reported, there was evidence of low usage of validated assessment tools. Therefore, based on this and the overall heterogeneity of included studies, the use of validated assessment tools would not appear to be widespread. Compared to non-validated assessment tools, validated assessment tools result in more accurate delirium recognition and greater improved outcomes for patients (Devlin et al., 2018). Therefore, the use of validated assessment tools is recommended in guidelines such as the Society of Critical Care Medicine’s delirium guidelines, an evidence-based guide for patient care in the intensive care unit (Devlin et al., 2018). Early recognition of delirium is one of the safest strategies for delirium management, and this can be improved through the use of validated delirium assessment tools (Sanga and Ansryan, 2025). For the most effective management, assessment tools should be used with validated predictive scores, such as the ‘Prediction of Delirium in ICU Patients (PRE-DELIRIC) score’. This can identify patients at high risk for developing delirium ahead of time (Gravante et al., 2023). The sooner delirium is recognised, the sooner interventions and preventative measures can be implemented, reducing the negative outcomes associated with delirium (Richly et al., 2025). A quality and safety project found that after implementing routine delirium assessments using the Short Confusion Assessment Method, patients with delirium had a reduced length of stay. Additionally, the percentage of falls that were associated with delirium also reduced (Vonnes and Tofthagen, 2022). Low validated assessment tool usage has negative implications for clinical practice, and the results of this review indicate that this should be addressed in order to improve delirium management.

While validated assessment tools are not widely used, other forms of assessment are used more frequently. Psychiatric consultations were the most common form of assessments that did not involve validated assessment tools. A psychiatric evaluation is the gold-standard for diagnosing delirium. However, as this can only be performed by trained physicians, it is not a first line option in many cases, hence the importance of validated delirium assessment tools (Lin et al., 2023). Other forms of frequently used assessments can be divided into personal judgement and unsuitable tools. Methods identified as personal judgement include clinical judgement, assessing the patient’s ability to follow commands, and evaluation of agitated related events. Unsuitable tools refer to tools that while validated, are not validated for the assessment of delirium, such as the Glasgow Coma Scale and the Richmond Agitation-Sedation Scale. While both of these tools support the assessment of delirium, it is not recommended for them to be used as the sole diagnostic tool (Trivedi and Iyer, 2016). Clinical Institute Withdrawal Assessment, Alcohol, Revised measures the severity of alcohol withdrawal symptoms and is commonly used to assess for delirium tremens. Delirium tremens is a severe form of alcohol withdrawal which only impacts a very small proportion of the population and therefore the Clinical Institute Withdrawal Assessment of Alcohol Scale, Revised is not applicable for the vast majority of delirium assessments (Grover and Ghosh, 2018). The use of these tools to assess delirium reflect the reported lack of delirium knowledge. The reported misbelief that delirium tools are unnecessary is also reflected in the use of unsuitable tools. The most commonly used validated assessment tool reported was the Confusion Assessment Method for the Intensive Care Unit. This is likely influenced by the large number of studies that were conducted in critical care units. The Intensive Care Delirium Screening Checklist, another tool used specifically in critical care settings, was also used frequently. Both tools are valid and reliable in the assessment of delirium in the critical care units, with the Confusion Assessment Method for the Intensive Care Unit being considered the superior of the two (Chen et al., 2021). The Confusion Assessment Method, Nursing Delirium Screening Scale, and 4AT were frequently used. These tools are not designed specifically for a critical care setting and are also valid and reliable for the assessment of delirium (Lin et al., 2023).

This scoping review set out to identify the barriers nurses report to using validated delirium assessment tools. Multiple barriers were related to the tools themselves. These barriers may stem from a lack of training and education. Tools seeming difficult or complex indicate inadequate training. Many assessment tools are designed to be rapid (Liu et al., 2023). Despite this, nurses report the tools are too time consuming. This again, reflects a potential lack of training and lack of awareness regarding the value of the assessment. Tools not being considered useful or trustworthy can stem from a lack of understanding about the tool. Educational interventions improve nurses’ positive relationship with tools, indicating that a lack of education causes a negative relationship (Zhao et al., 2024). Opportunities to improve the efficiency and effectiveness of delirium assessment tools should always be considered. However, many of the barriers nurses encounter that relate to the tools more likely stem from a lack of awareness and understanding, particularly when put into context with the other barriers.

Multiple studies mentioned nurses’ lack of delirium and assessment tool knowledge as a barrier to use as well as a lack of confidence administering the assessment tools. This mirrors the literature which indicates that there is knowledge and confidence deficiency in nurses when it comes to delirium care (Sinvani et al., 2016; Waszynski et al., 2024). Confidence in delirium assessment is closely associated with delirium knowledge (Zhao et al., 2024). The provision of education and training is likely to remove multiple barriers at the individual nurse level. Barriers at the individual nurse level were less likely to be reported than other barriers. Future researchers and policy makers aiming to address barriers should note that nurses’ may be disinclined to recognise their own gaps in knowledge and attitudes.

Patients were referred to frequently as being a barrier to delirium assessment. Primarily these barriers came in the form of patient factors such as patients who are mechanically ventilated or patients who did not speak the same language as the nurses. Some of these barriers outline the lack of knowledge and training nurses have received as some tools. For example, the Confusion Assessment Method for the Intensive Care Unit and Intensive Care Delirium Screening Checklist can be used in mechanically ventilated patients and during sedation stops (Haenggi et al., 2013). Other barriers, such as language differences and deaf patients, highlight existing gaps in assessment tools. While tools such as the 4AT can suggest the presence of delirium from observation alone, communication is still necessary for confirmation (Alhaidari and Matsis, 2022). Other barriers at the patient level relate to nurses not wanting to burden their patient. The questions used in the Confusion Assessment Method for the Intensive Care Unit are considered to be condescending to patients and can cause a patient stress should they get them incorrect. These concerns may indicate a lack of training as nurses are not comfortable explaining the test to their patients in a way that would remove any stigma. While nurses may omit the tests to protect their patient, patients can also resist taking the test. Uncooperative patients are to be expected when assessing for delirium, particularly if a patient has hyperactive delirium. As mentioned, while observation alone can indicate delirium, tools still require communication with the patient. Further training and hospital policies would be required to address patients who are unwilling to partake in a test.

Barriers at the organisational level were most commonly cited by nurses. A specific barrier regularly discussed was other clinicians not valuing the assessment tool findings. The majority of the time this referred to physicians. Due to the findings not being acted on, nurses did not see delirium assessment tools as a valuable use of time. A 2016 study found that while physicians had a higher delirium knowledge score than nurses, they were less likely to consider the treatment of delirium as extremely important (Sinvani et al., 2016). This indicates delirium being disregarded at a hospital-wide level and stresses the importance of a multi-clinician approach. Additionally, nurses feature how they lacked the support from their hospital to use assessment tools. This came in the form of not receiving education, training, or protocols for the use of the tool. Specifically, nurses highlighted their difficulty establishing a patients’ baseline mental status. The measuring and recording of the baseline should occur on admission to the hospital with the use of a validated tool or speaking with a knowledgeable informant. This is crucial in delirium assessment as it necessary for an acute change to be noticed. Additionally, it is necessary for acute differential diagnosis from other conditions such as dementia or depression (Oh et al., 2017). Though baseline mental status is frequently measured by nurses, it is not often documented effectively, resulting in inadequate reporting of mental status (Steis and Fick, 2008). Protocols for this data collection will inevitably support nurses in their delirium assessments.

In addition to barriers, this review aimed to identify the facilitators to using validated delirium assessment tools. Opportunities to learn were the most commonly identified facilitator by nurses. Nurses report the provision of education, practice, and training as a key element in their ability to use assessment tools, as does the existence of a protocol. These facilitators directly correspond with the organisational barriers identified by nurses. It has been proven that educational interventions improve nurses’ knowledge of delirium, their confidence in managing the syndrome, and their overall compliance with delirium assessments (Zhao et al., 2024). Globally, the existence of a delirium management protocol is associated with the use of validated delirium assessment tools (Nydahl et al., 2024).

Tools that are considered easy and efficient to use are facilitators to use. Many of the validated assessment tools are short in length which contributes to this (Liu et al., 2023). Nurses also outlined the importance of integrating the assessment tool into their current practice, either through existing charts or assessments or within the electronic documentation system. However, as previously mentioned, for a tool to be considered quick, easy, or effective, training and education are required.

Having pre-existing awareness and understanding of delirium is considered a facilitator. In many cases, nurses first are introduced to delirium on their undergraduate nursing courses. While there is little global research done on this topic, an evaluation of Scottish nursing undergraduate programmes found that while all include delirium education, the content and quality of said content is extremely varied (Copeland and Barron, 2020). This produces nurses with varying levels of delirium awareness and understanding working with patients in acute care. While the provision of delirium education at the undergraduate level needs to be improved, hospitals equally must be aware of potential inadequate knowledge levels among graduate nurses and provide the necessary opportunities to improve their knowledge.

Hospitals where the baseline mental status is assessed allows for nurses to assess for delirium with greater accuracy. A defining characteristic of delirium is an acute change in attention and awareness (*Diagnostic and statistical manual of mental disorders : DSM-5.* Fifth edition, 2013). Assessment tools require nurses to note if there has been a recent change in the patient’s mental status, and for this to be successful, nurses must be aware of the patient’s baseline mental status. It has been established that screening cognitive baseline on admission is of great importance, yet is not always done effectively (Steis and Fick, 2008). Enforcing protocols that require cognitive assessments on admission would rectify this concern.

The majority of barriers reported by nurses relate to organisational problems. Conversely, nurses report that having a positive staff culture towards delirium assessment can act as a facilitator. The importance of hospital-wide initiatives has been discussed previously. Positive staff culture also references the importance of guidance and support from leadership when it comes to delirium assessment tools. At a general level, positive nursing leadership is associated with increased nursing expertise, increased collaboration, and lower staff turnover. It is also associated with a decrease in adverse events and mortality for patients as well as increasing patient satisfaction (Wong et al., 2013). Having positive leadership that promotes delirium assessment tools to all clinicians has a likelihood of positively impacting nurses’ use of validated delirium assessment tools.

### 4.1 Limitations

To ensure that the search for sources was comprehensive, four databases were searched in addition to grey literature sources. Forwards and backwards citation searching were also undertaken for all included sources. To prevent bias, both rounds of screening were completed independently by two separate researchers. However, there are limitations to this scoping review. Only English sources were included, creating language bias. As many studies were conducted in non-English speaking countries such as China, there is a likelihood that relevant studies have been published in other languages and have not been included in this review. Despite being conducted under the guidance of an experienced librarian, there is the possibility that the search strategy did not capture every relevant source. Any negative impact of this should have been mitigated by the forward and backward citation searching. This scoping review purposefully excluded the validated tool usage rates from intervention studies. As the rates in these studies are directly influenced by the intervention, they are not reflective of similar units in similar contexts. While this ensures the results in this review are generalisable, the review does not capture the impact interventions have on rates.

### 4.2 Conclusions

Usage of validated delirium assessment tools among nurses in general acute care settings is generally low. However, due to extreme heterogeneity among studies, widespread usage cannot be determined. The Confusion Assessment Method for the Intensive Care Unit is the most commonly reported validated tool within the studies; however, this is likely a reflection of the majority of studies taking place within critical care units. Organisational level barriers were most commonly identified among nurses when discussing barriers to using validated delirium assessment tools while opportunities to learn were the most cited facilitators. It is clear from this scoping review that there has been an increased interest on the topic of nurses' use of delirium assessment tools in recent years. However, a significant gap remains between best practice guidelines and the reality of clinical practice. At a hospital policy level, identified barriers must be removed and identified facilitators must be implemented. At a research level, the impact of these changes must be investigated in order to identify the most effective way of promoting assessment tool usage. The provision of education and training is clearly highlighted within the literature as being an essential influence on the use of assessment tools and this should be the first step taken by all parties.

## Data Statement

No data is associated with this article.

## CRediT authorship contribution statement

**Aoibhinn Boyd:** Writing – original draft, Visualization, Methodology, Investigation, Formal analysis, Data curation, Conceptualization. **Marcia Kirwan:** Writing – review & editing, Supervision, Project administration, Funding acquisition. **Leona Bannon:** Writing – review & editing, Validation, Supervision, Project administration, Funding acquisition.

## Declaration of competing interest

The authors declare the following financial interests/personal relationships which may be considered as potential competing interests: Given her role as Associate Editor at International Journal of Nursing Studies Advances, Leona Bannon had no involvement in the peer-review of this article and has no access to information regarding its peer-review. Full responsibility for the editorial process for this article was delegated to another journal editor. If there are other authors, they declare that they have no known competing financial interests or personal relationships that could have appeared to influence the work reported in this paper.

## Appendix Supplementary materials

Image, application 1

Image, application 2

Image, application 3

Image, application 4
