# Supplementary material for: Nurses’ usage of validated tools to assess for delirium in general acute care settings: A scoping review
Source: Int J Nurs Stud Adv. 2026 May 26;11:100579. doi: 10.1016/j.ijnsa.2026.100579 (PMC13265650; doi:10.1016/j.ijnsa.2026.100579)
Supplement: Supplementary file 1 [file mmc1.docx]

Data Extraction Tool

| First Author |
| --- |
| Other Authors |
| Year of Publication |
| Country of Origin |
| Item Type |
| Study Title |
| Study Methodology |
| Study Design |
| Aim of Study |
| Primary Outcomes |
| Secondary Outcomes |
| Method of Data Collection |
| Country Study was Conducted |
| How Many Study Sites |
| Type of Ward(s) |
| Nurse Sample Size |
| Delirium Assessments Investigated |
| Percentage of nurses who use validated assessment tools |
| Barriers identified |
| Facilitators identified |
