# Supplementary material for: Nurses’ usage of validated tools to assess for delirium in general acute care settings: A scoping review
Source: Int J Nurs Stud Adv. 2026 May 26;11:100579. doi: 10.1016/j.ijnsa.2026.100579 (PMC13265650; doi:10.1016/j.ijnsa.2026.100579)
Supplement: Supplementary file 3 [file mmc3.docx]

Barriers to delirium assessment tool use

| Theme | Concept | Barrier | Sources |
| --- | --- | --- | --- |
| **Tool Level** |  |  |  |
|  | **Difficulty and complexity of tool** |  |  |
|  |  | Too complex | Anbu 2014  Biyabanaki 2020  Devlin 2008  Meghani 2024  Özsaban 2016  Ramoo 2018  Rowley-Conwy 2017 |
|  |  | Too difficult | Emme 2020  HallbergKristensen 2024 |
|  |  | More difficult than unstructured assessments | Reade 2011 |
|  | **Length of tool** |  |  |
|  |  | Too long | Oxenbøll-Collet 2018 |
|  |  | Time consuming | HallbergKristensen 2024  Meghani 2024  Ramoo 2018 |
|  | **Mistrust in tool** |  |  |
|  |  | Considered unreliable | Alhaidari 2017  Reade 2011 |
|  |  | Lack of confidence in tool | Steinseth 2018  Zamoscik 2017 |
|  |  | Mistrusts the results | Oxenbøll-Collet 2018  Steinseth 2018 |
|  | **Tool is considered unnecessary** |  |  |
|  |  | Deemed unnecessary for experienced nurses | Oberai 2019 |
|  |  | Not comprehensive enough | Aldwikat 2023  Oberai 2019 |
|  |  | Existing documentation is sufficient | Gao 2022  Oberai 2019  Swan 2011 |
|  |  | Not deemed valuable | Steinseth 2018 |
|  |  | Too subjective | Aldwikat 2023  Ragheb 2023 |
| **Nurse Level** |  |  |  |
|  | **Lack of knowledge and skills** |  |  |
|  |  | Lack of knowledge on tool | Correya 2025  dosSantos 2022  Meghani 2024  Soja 2008  Zhou 2023 |
|  |  | Lack of delirium knowledge | dosSantos 2022  Emme 2020  Fraser 2018  Wong 2018 |
|  |  | Lack of competence to use tool | Gao 2022  Zhou 2023 |
|  |  | Difficulty differentiating between delirium and other conditions | dosSantos 2022  Wong 2018  Zamoscik 2017 |
|  | **Nursing beliefs** |  |  |
|  |  | Not a priority | Emme 2020  Oxenbøll-Collet 2018  Zamoscik 2017 |
|  |  | Lack of self-confidence | Andrews 2015  Biyabanaki 2020  Kandindi 2018  Lange 2023  Oxenbøll-Collet 2018 |
|  |  | It is not the nurses’ role to diagnose | Swan 2011  Wong 2018 |
|  |  | Deemed unnecessary for patients who arrive with delirium | Oberai 2019 |
| **Patient Level** |  |  |  |
|  | **Fear of harming patient** |  |  |
|  |  | False positives can have negative outcomes for the patient | Oxenbøll-Collet 2018  Swan 2011 |
|  |  | Can embarrass patient | Oxenbøll-Collet 2018 |
|  |  | Considered to be condescending to patients | Steinseth 2018 |
|  |  | Disrupts caregiving relationship | Oxenbøll-Collet 2018 |
|  |  | Burdens the patient | Aldwikat 2023  Jung 2013  Oxenbøll-Collet 2018 |
|  |  | Repeated tests negatively impact the patient | Jung 2013  Reppas-Rindlisbacher 2021 |
|  | **Patient factors make tool harder to complete** |  |  |
|  |  | Influence of drugs on patient cognition | Swan 2011 |
|  |  | Patients with poor health literacy | Reppas-Rindlisbacher 2021 |
|  |  | Difficulty of use with deaf patients/patients with hearing loss | Aldwikat 2023  Reppas-Rindlisbacher 2021 |
|  |  | Difficulty of use in intubated patients | Lunghi 2012  Ramoo 2018  Scott 2013  Trask 2021 |
|  |  | Difficulty of use in sedated patients | Aldwikat 2023  Lunghi 2012  Oxenbøll-Collet 2018  Scott 2013  Zamoscik 2017 |
|  |  | Difficulty of use in patients receiving mechanical ventilation | Andrews 2015  dosSantos 2022 |
|  |  | Difficulty of use with patients with tracheostomy | Aldwikat 2023 |
|  |  | Unreliable in depressed patients or patients in pain | Zamoscik 2017 |
|  |  | Language barriers | Aldwikat 2023  Correya 2025  Jung 2013  Reppas-Rindlisbacher 2021  Wong 2018 |
|  | **Patient and families disrupt the use of the tool** |  |  |
|  |  | Patients resist assessment | Aldwikat 2023  Correya 2025  dosSantos 2022  Emme 2020  Zamoscik 2017 |
|  |  | Repeated tests allow for memorisation | Zamoscik 2017 |
|  |  | Family members cause nurse discomfort | Oxenbøll-Collet 2018 |
| **Organisational level** |  |  |  |
|  | **Negative culture towards tools** |  |  |
|  |  | Lack of effort from leadership | Ragheb 2023 |
|  |  | Staff resistant to change | Correya 2025 |
|  |  | Findings aren’t valued by other staff | Andrews 2015  HallbergKristensen 2024  Jung 2013  Law 2012  Oberai 2019  Oxenbøll-Collet 2018  Ragheb 2023  Ramoo 2018  Scott 2013  Wong 2018  Zamoscik 2017 |
|  |  | Lack of feedback on performance | Law 2012  Soja 2008 |
|  | **Hospital environment** |  |  |
|  |  | Masks and gloves hinder communication | Jung 2013 |
|  |  | Unsuitable interpretation services | Reppas-Rindlisbacher 2021 |
|  |  | Background noise | Reppas-Rindlisbacher 2021 |
|  | **Nurses are at their capacity** |  |  |
|  |  | Time constraints | Aldwikat 2023  HallbergKristensen 2024  Law 2012  Reppas-Rindlisbacher 2021  Soja 2008  Wong 2018  Zamoscik 2017 |
|  |  | Existing workload is too high | Ragheb 2023 |
|  |  | Too much existing paperwork | Emme 2020  Swan 2011 |
|  | **No organisational supports for the use of tools** |  |  |
|  |  | Hard to establish baseline | Aldwikat 2023  Emme 2020  Ragheb 2023  Reppas-Rindlisbacher 2021  Swan 2011  Wong 2018 |
|  |  | Lack of protocol | Gao 2022  Ragheb 2023  Zhou 2023 |
|  |  | Not routine | Steinseth 2018 |
|  |  | No tool provided | Gao 2022  Zhou 2023 |
|  |  | Lack of subtype assessments | Zhou 2023 |
|  |  | Lack of training | Carin-Levy 2013  Correya 2025  dosSantos 2022  Alhaidari 2017  Ragheb 2023  Steinseth 2018 |
|  |  | Lack of education | Ragheb 2023  Steinseth 2018 |
