# Supplementary material for: Nurses’ usage of validated tools to assess for delirium in general acute care settings: A scoping review
Source: Int J Nurs Stud Adv. 2026 May 26;11:100579. doi: 10.1016/j.ijnsa.2026.100579 (PMC13265650; doi:10.1016/j.ijnsa.2026.100579)
Supplement: Supplementary file 4 [file mmc4.docx]

Facilitators to delirium assessment tool use

| Theme | Facilitator | Source |
| --- | --- | --- |
| **Ease and efficiency of tool** |  |  |
|  | Tool is considered thorough | Aldwikat 2023 |
|  | Tool is easy to use | Aldwikat 2023  Emme 2020 |
|  | Easy access within existing charts | Andrews 2015 |
|  | Electronic documentation system | Wong 2018 |
|  | Availability of note cards | Andrews 2015 |
|  | Tool is time efficient | Aldwikat 2023  Emme 2020 |
| **Improved understanding of delirium** |  |  |
|  | Increased awareness of delirium | Correya 2025 |
|  | Understanding the benefits of the tool | Oberai 2019 |
|  | Having existing knowledge about delirium | Fraser 2018 |
| **Establishing baseline mental status** |  |  |
|  | Pre-screen to establish baseline | Swan 2011 |
|  | Involvement of family | Ragheb 2023  Reppas-Rindlisbacher 2021 |
| **Opportunities to learn** |  |  |
|  | Existence of a protocol | Ragheb 2023  Zamoscik 2017 |
|  | Familiarity with tools | Correya 2025 |
|  | Education | Andrews 2015  Ragheb 2023 |
|  | Training | Steinseth 2018  Swan 2011  Zamoscik 2017 |
|  | Practice | Correya 2025  Jung 2013 |
| **Positive staff culture** |  |  |
|  | Culture of open-mindness | Steinseth 2018 |
|  | Encouragement from leadership | Correya 2025 |
|  | Support from other staff | Steinseth 2018 |
|  | Guidance from leadership | Aldwikat 2023  Steinseth 2018 |
